# Akt finds its new path to regulate cell cycle through modulating Skp2 activity and its destruction by APC/Cdh1

**DOI:** 10.1186/1747-1028-4-11

**Published:** 2009-06-23

**Authors:** Daming Gao, Hiroyuki Inuzuka, Alan Tseng, Wenyi Wei

**Affiliations:** 1 Department of Pathology, Beth Israel Deaconess Medical Center, Harvard Medical School, Boston, MA 02215, USA

## Abstract

Skp2 over-expression has been observed in many human cancers. However, the mechanisms underlying elevated Skp2 expression have remained elusive. We recently reported that Akt1, but not Akt2, directly controls Skp2 stability by interfering with its association with APC/Cdh1. As a result, Skp2 degradation is protected in cancer cells with elevated Akt activity. This finding expands our knowledge of how specific kinase cascades influence proteolysis governed by APC/Cdh1 complexes. However, it awaits further investigation to elucidate whether the PI3K/Akt circuit affects other APC/Cdh1 substrates. Our results further strengthen the argument that different Akt isoforms might have distinct, even opposing functions in the regulation of cell growth or migration. In addition, we noticed that Ser72 is localized in a putative Nuclear Localization Sequence (NLS), and that phosphorylation of Ser72 disrupts the NLS and thus promotes Skp2 cytoplasmic translocation. This finding links elevated Akt activity with the observed cytoplasmic Skp2 staining in aggressive breast and prostate cancer patients. Furthermore, it provides the rationale for the development of specific Akt1 inhibitors as efficient anti-cancer therapeutic agents.

## Introduction

In dividing cells, the cell cycle is tightly controlled by multiple regulatory mechanisms to ensure that DNA is faithfully replicated only once in the S phase and then distributed equally between two daughter cells in the M phase. Defective cell cycle regulation can lead to genomic instability, which ultimately facilitates cancer development. Many key regulators governing the cell cycle progression are short-lived proteins, and selective degradation of these regulators by the ubiquitin-proteasome system has recently been shown to be a major mechanism for ensuring ordered and coordinated cell cycle progression [1,2]. Moreover, the irreversible nature of proteolysis guarantees the uni-directional execution of the cell cycle program, driving the cell cycle from one stage to the next. There are two related, multi-subunit E3 ubiquitin ligase enzymes, the Anaphase Promoting Complex (APC) and the Skp1-Cullin1-F-box complex (SCF) that are considered to be the major driving forces governing proper cell cycle progression [3]. SCF is active from the late G1 phase until the G2 phase and mediates the ubiquitination of G1 cyclins and Cdk inhibitors. SCF consists of the invariable components Skp1, Cul1, and Rbx1, as well as a variable component known as an F-box protein that is responsible for substrate recognition. There are 68 putative F-box proteins encoded in the human genome which can form individual SCF complexes, each with different F-box proteins incorporated into the core Skp1/Cullin-1-Rbx1 complex [4]. The diversity of these SCF complexes ultimately provides the high stringency necessary for substrate specificity. The well-characterized F-box proteins Skp2, Cdc4/Fbw7, and β-Trcp1 target p27 [5], cyclin E [6,7], and Cdc25A [8,9], respectively, for ubiquitination and degradation. In all cases, proper phosphorylation of the substrate is required for interaction with the F-box proteins. Unlike SCF, APC is active from the late G2 phase to the mid-G1 phase, and is responsible for the degradation of mitotic cyclins, securin, and geminin. Although APC is composed of 11 subunits, the general structure is very similar to SCF. The substrate adaptors Cdc20 and Cdh1 are equivalent to the F-box proteins, but both Cdh1 and Cdc20 do not require post-translational modification of their respective substrates for recognition, instead, binding to their substrates via Destruction Boxes (D-Box) or KEN Boxes.

Skp2 was originally identified as an S-phase Kinase Cdk2/Cyclin A-associated protein [10]. Subsequently, the identification of an F-box domain within its coding sequence suggested the presence of E3 ubiquitin ligase activity [5,11]. Besides its major downstream target p27, recent studies have demonstrated that the Skp2/SCF complex also targets numerous other substrates for degradation, many of which are negative cell cycle regulators. These include p21, p57, p130 and FOXO1 [1]. p27 functions as a tumor suppressor such that its inactivation predisposes mice to cancer development [12]. However, in contrast with known tumor suppressor genes such as p53 or Rb, homozygous loss or silencing of the p27 gene is rarely found in human cancers. Instead, it is reduced p27 protein expression which is often linked to human malignancy, suggesting that regulation occurs mainly at the post-translational level [13]. Indeed, elevated Skp2 expression is frequently observed in many tumors including breast and prostate carcinomas [14,15]. It has been proposed that enhanced Skp2 expression leads to the accelerated degradation of targets such as p27 and other cell cycle regulators, thus promoting cell cycle progression and favoring transformation. Furthermore, overexpression of Skp2 facilitates transformation of Rat1 cells in soft agar and in nude mouse xenografts [14]. The oncogenic potential of Skp2 is further illustrated in transgenic mice. In one report, overexpression of Skp2 in the mouse prostate induced hyperplasia, dysplasia and low-grade carcinoma [16], while others have reported that Skp2 transgenic mice co-expressing N-Ras develop lymphomas [17]. These findings support the contention that Skp2 overexpression inversely correlates with low p27 expression, and positively correlates with tumor malignancy and poor diagnosis.

However, the molecular mechanisms underlying elevated Skp2 expression have not been fully explored. We and others have previously demonstrated that Cdh1 is the upstream E3 ubiquitin ligase which promotes Skp2 destruction [18,19]. In contrast to the frequency of Skp2 overexpression, loss of Cdh1 is not a frequent event in human cancer. Thus, loss of Cdh1 cannot explain the observation of elevated Skp2 levels in carcinomas. On the other hand, hyperactivation of the Akt pathway through various means of genetic alterations is considered a hallmark of many cancers. Furthermore, it has been reported that activation of the PI 3-K (phosphoinositide 3-kinase)/Akt pathway enhances p27 destruction [20]. This suggests that sustained Akt activity can influence Skp2 activity. Consistent with this, studies have also demonstrated that Akt can contribute to Skp2 overexpression, although the mechanism has not been explored [21,22].

The Akt family of kinases is composed of three closely related family members designated Akt1, Akt2 and Akt3, also known as PKBα (Protein Kinase B), PKBβ and PKBγ, respectively. Akt isoforms are known to play critical roles in many cellular processes including proliferation, transformation, survival and metabolism [23]. The PI 3-K and Akt pathway is frequently amplified and hyperactivated in most human cancers [24]. Upon activation of receptors for growth factors such as IGF-1 (insulin-like growth factor-1), activation of PI 3-K leads to the synthesis of the second messenger PtdIns-3,4,5-P_3 _which binds and recruits Akt to the plasma membrane. Phosphorylation of Akt by upstream kinases fully activates the enzyme allowing it to phosphorylate multiple substrates which contain a minimal motif usually comprising RxRxxS/T (hereby x is any amino acid) [23]. Akt activity is negatively regulated by two tumor suppressors, the PTEN lipid phosphatase which dephosphorylates PtdIns-3,4,5-P_3 _[25], and PHLPP, a Ser/Thr phosphatase which dephosphorylates Akt at Ser473, leading to its inactivation [26]. Since most of the upstream regulators and downstream mediators of the Akt pathway are either oncogenes or tumor suppressors, it is not surprising to find that Akt activity is abnormally elevated in most human cancers [27]. Major mechanisms described to date which explain hyperactivation of Akt include loss-of-function mutations in PTEN, as well as gain-of-function mutations in upstream regulators such as the receptors HER2, EGF-R and Ras [28]. In addition, constitutively active PI 3-K mutations have been identified in several human cancers and shown to be causally linked to elevated Akt signaling [29]. Enhanced Akt signaling in tumor cells can suppress apoptosis by promoting the phosphorylation and subsequent cytoplasmic localization of many downstream pro-apoptotic target proteins such as Bad [30], FOXO1 [31] and FOXO3a [32]. Akt upregulation can also promote cell growth by inactivating the negative cell cycle regulators p21 [33] and p27 [34-36]. Most studies that have explored a role for the PI 3-K and Akt pathway in cell cycle progression, survival and cancer progression have generally assumed that all three isoforms function in a overlapping and redundant manner. However, recent studies have begun to suggest isoform-specific functions for Akt. This was first highlighted by distinct phenotypes born out from the Akt1 and Akt2 knockout mice [37]. At the level of signaling and cell cycle progression, Akt1 has been shown to promote cell cycle progression, whereas Akt2 promotes cell cycle exit in myoblasts [38]. The PHLPP1 and PHLPP2 isoforms differentially dephosphorylate Akt1 and Akt2 leading to distinct accessibility of each Akt isoform to substrates such as p27, FOXO3a and GSK-3 [39,40]. Finally, Akt1 and Akt2 have been shown to function in an opposing manner in the regulation of breast cancer cell invasive migration. (Irie et al., 2005).

## The Skp2 Ser72 Akt phosphorylation site is conserved in large mammals

**Figure 1:**
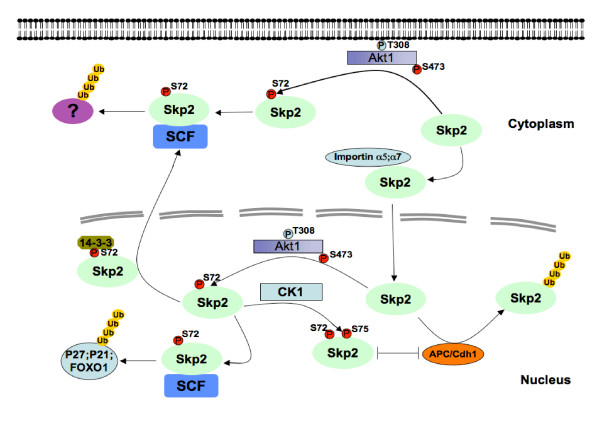
**Schematic model for how Akt1-dependent phosphorylation of Skp2 at the Ser72 site promotes Skp2 cytoplasmic localization and stabilizes Skp2 by impairing its association with the APC/Cdh1 E3 ubiquitin ligase complex**. Phosphorylation of Skp2 at Ser72 by Akt1 greatly reduces its ability to interact with the importin complex as well as promotes its association with 14-3-3, resulting in cytoplasmic retention. Furthermore, phosphorylation of Skp2 by Akt1 primes Skp2 for subsequent phosphorylation of Ser75 by Casein Kinase I (CKI). Phosphorylation on both Ser72 and Ser75 results in impaired association with Cdh1, thus allowing Skp2 to escape APC/Cdh1-mediated ubiquitination and destruction.

Following these clues, we and others recently demonstrated that elevated Akt activity could positively influence Skp2 activity, and impair its destruction by the APC/Cdh1 E3 ubiquitin ligase complex [41,42] (Figure 1). At first glance, this offers a molecular mechanism for the frequently observed Skp2 overexpression in many human cancers and links this phenomenon to the abnormal activation of the PI 3-K and Akt pathway, which is well-documented as being aberrantly activated in the majority of human cancers. The Ser72 site is conserved in most large mammals we examined, including primates, dogs, horses, pigs, cows, and even rats. However, this putative Ser72 Akt phosphorylation site is not present in the mouse sequence. It is known that similar inter-species differences also exist for other Akt substrates including p27 [36] and caspase-9 [43]. We further found that the Ser72 site is not conserved in Xenopus or Zebrafish, thus indicating that the Akt/Skp2 regulatory pathway might have evolved as a gain-of-function event later in evolution. On the other hand, it is well-established that tumorigenesis differs dramatically between humans and mice [44]. Hence, it is plausible that for large animals with a longer life span than mice, which requires more cell division events, an additional layer of cell cycle control is developed. It is also possible that there is another universal molecular mechanism shared by most species to control Skp2 stability/activity, which is not identified yet.

## The potential role for Casein Kinase I in Skp2 stability control

Our results further demonstrate that phosphorylation of human Skp2 at Ser72 creates a priming site, and that CKI may be one of the kinases which phosphorylates Ser75, a process that would lead to subsequent dissociation from Cdh1 and stabilization of Skp2. Sequence analysis also reveals that the Ser75 site is not conserved in all mammals we examined; although most large mammals contain Ser72, they do not contain Ser75, which is replaced by an Asn. This indicates that CKI or other Ser75 kinases are not likely to efficiently phosphorylate Skp2 in these species. Clearly additional studies are required to examine in more detail the regulation of Ser75 phosphorylation in human Skp2, and to determine if CKI is the physiologically-relevant kinase, and similarly whether Akt regulates Skp2 stability in species that do not harbor Ser75.

Regardless, our data clearly points to an important distinction between human and mouse Skp2 regulation by the Akt pathway. First, phosphorylation of Ser72 is critical for the ability of Akt to regulate Skp2 stability. Since mouse Skp2 does not contain this site, it explains why mouse Skp2 expression is not affected by the Akt pathway. Secondly, additional experiments using human Skp2 have revealed distinct contributions of phosphorylation events to Skp2 stability. Phosphorylation of Ser72 by Akt is sufficient to disrupt the association between importin and Skp2, leading to Skp2 cytoplasmic translocation. Moreover, phosphorylation of both Ser72 and Ser75 by Akt1 and CKI, respectively, is required to disrupt the association between Cdh1 and Skp2, thus stabilizing Skp2. However, since Cdh1 is primarily localized in the nucleus, phosphorylation of Skp2 by Akt itself might be sufficient to stabilize a portion of Skp2 by cytoplasmic translocation.

## Akt1 regulates both Skp2 stability and Skp2 transcription

Our data argues that the function of Akt at regulating Skp2 levels is primarily through the regulation of Skp2 protein stability. Substitution of the Ser72 residue to a non-phosphorylatable Ala created a much more unstable Skp2 protein. Conversely, the phospho-mimetic S72D/S75D Skp2 mutant is much more stable than the wild-type protein. This is likely due to the ability of Akt to affect Cdh1-mediated Skp2 degradation since the S72D/S75D mutant resists degradation, and because Akt failed to protect the S72A mutant from degradation. Moreover, in cells where Akt1 is depleted, reduced phosphorylation of Ser72 in Skp2 correlates with a marked decrease in Skp2 levels. This process is likely due to enhanced Skp2 degradation by Cdh1, since inactivation of Cdh1 resulted in restoration of Skp2 to a level comparable to control siRNA-treated samples. Our results do not necessarily disagree with a recent report which showed that activation of PI 3-K and Akt also influences Skp2 mRNA levels [45]. It has been shown that Skp2 is a downstream target of E2F-1 [46], and thus the regulation of its expression at the level of transcription is higher in cells in which Rb is defective. It has also been reported that activation of Akt promotes the binding of E2F-1 to the proximal Skp2 promoter [47]. Therefore, Skp2 upregulation in most human cancers might be due to a synergistic action of upregulated Skp2 mRNA levels with a concomitant evasion of Cdh1-mediated degradation.

## Does Akt regulate other APC/Cdh1 substrates other than Skp2?

For most SCF/F-box complexes, the regulation of substrate recognition occurs at the level of the substrate, such that the F-box protein will usually not recognize its downstream substrates without a specific combination of phosphorylation events. On the other hand, the interaction of Cdh1 and Cdc20 with their substrates usually does not require any post-translational modifications [48]. In this case, the regulation of APC activity occurs primarily on the APC complex itself. Phosphorylation of Cdc20 by Plk and Cdc2/Cyclin B is required for the activation of the APC/Cdc20 complex while phosphorylation of Cdh1 by the Cdk2/Cyclin A complex terminates Cdh1 activity by dissociating Cdh1 from the APC core subunits [49]. Recently, it was shown that APC/Cdh1 ubiquitinates and degrades its substrates with different kinetics, and the preference of degradation order depends on the relative processivity of substrate multiubiquitination by APC/Cdh1 [50].

Our finding provides another unique mechanism for the selective degradation of Cdh1 downstream targets. In all the known Cdh1 substrates tested so far, only Skp2 expression levels are affected by Akt signaling. This provides a novel link between p27 degradation and Akt activation, both of which can be induced by growth factors. Unlike most E3 ubiquitin ligases, Cdh1 is active in a low-kinase state during early G1 and in quiescent cells. Thus it is assumed that Cdh1 plays a major role in the maintenance of quiescence [51]. It is also known that most cells in human tissues are in a differentiated (post-mitotic) state, and in many cases loss of this differentiated state plays a major role in promoting tumorigenesis, and p27 has been demonstrated to be a critical player. KPC has been shown to play an important role in triggering the degradation of p27 in the early G1 phase [52]. Furthermore, Skp2-governed p27 degradation is critical for the timely activation of Cdk2 activity, and subsequent transition through the restriction point. Consistent with a previous report [5], our own studies show that activation of Akt upon serum stimulation allows for the early induction of Skp2, which correlates with p27 disappearance. This protective mechanism mediated by the Akt pathway is very similar to the Cdk2/cyclin E complex, which protects Cdc6 from Cdh1-mediated destruction [53]. Thus, our finding expands our knowledge of how specific kinase cascades could influence Cdh1-governed proteolysis. In contrast to the SCF complex, where phosphorylation triggers substrate recognition, we propose that a specific phosphorylation event by Akt, in addition to CKI, disables the degradation mediated by APC/Cdh1. Upon Cdk2/cyclin E phosphorylation, the interaction between Cdh1 and Cdc6 is reduced [53]. Similar to their finding, we found that the interaction between Skp2 and Cdh1 is also affected by Akt phosphorylation.

The next outstanding question is whether Akt could affect the stability of other APC/Cdh1 substrates. We found that manipulation of Akt activity does not affect the expression of other known Cdh1 substrates we examined, including Cdc20, Plk-1, Geminin, Cdc6, Securin, cyclin A and cyclin B [3,49]. These results indicate that Akt might only specifically affects the destruction of a subgroup of Cdh1 substrates including Skp2 and others that are still unidentified. We used the Scansite program to screen all known Cdh1 downstream targets, and found that except for Cdc25A and DNMT1, none of the other Cdh1 substrates contains the canonical Akt site. Therefore, it is interesting to further investigate whether depletion of Akt1 results in decreased expression of both Cdc25A and DNMT1. Furthermore, similar to what Akt does to Skp2, whether elevated Akt activity protects Cdc25A and DNMT1 from Cdh1-mediated destruction.

## Akt promotes Skp2 cytoplasmic translocation

The Akt pathway functions to promote both cell survival and cell growth by inactivating many of its downstream substrates [23]. Interestingly, by the same token, phosphorylation of Akt substrates usually results in their cytoplasmic translocation. In the case of p27, p21 and FOXO proteins, the Akt phosphorylation site is proximal to the Nuclear Localization Sequence (NLS) and when phosphorylated creates a binding site that can be recognized by the 14-3-3 proteins. Recruitment of 14-3-3 results in the masking of the NLS and subsequent cytoplasmic translocation [54]. We also observed an interaction of 14-3-3 with Skp2 in cells expressing activated Akt. However, Ser72 phosphorylation per se is not sufficient for recruitment of 14-3-3. Thus it is possible that 14-3-3 is recruited to Skp2 after additional Akt phosphorylation events, or indirectly through interaction with Akt. The NLS of Skp2 resembles that of SV40 T antigen, which is recognized by the importin complex, the only difference being that Skp2 harbors the Ser72 Akt site. We also demonstrated that deletion of the NLS results in cytoplasmic localization, indicating the requirement of this sequence for nuclear import. Moreover, *in vitro *biochemical analyses demonstrated that deletion of the NLS disrupts the association between Skp2 and importin, providing further evidence that the specific association between Skp2 and the importin complex requires the NLS. Alternatively, the phosphorylation of serine or threonine residues by specific kinases within the NLS could impair the interaction between the importin complex and the NLS [55,56]. In keeping with this notion, we also demonstrated that phosphorylation of human Skp2 by Akt at Ser72 greatly reduces the interaction between Skp2 and importin. It is possible that both of these mechanisms contribute to the cytoplasmic translocation of Skp2 subsequent to Akt phosphorylation. This process is likely to be important for Skp2 function because Akt phosphorylation of the Skp2 downstream targets p27, p21 and FOXO1 also promotes their cytoplasmic localization, thus terminating their function. Our findings add a new dimension to this model by suggesting that Akt-induced cytoplasmic translocation of Skp2 may lead to elevated degradation of its downstream targets by the cytoplasmic SCF/Skp2 complex. Lin et al suggested that cytoplasmic Skp2 plays an important role in promoting cellular motility [42]. It is well documented that cytoplasmic Skp2 is frequently observed in more advanced breast and prostate cancer. Therefore, it is plausible that cytoplasmic Skp2 activity promotes metastasis. It is important to further elucidate the novel substrates for cytoplasmic Skp2, which will provide important insight for developing new anti-cancer treatments.

## Akt isoform specificity in the regulation of Skp2 protein stability

Interestingly, our data points to Akt isoform specificity in the regulation of Skp2 protein stability. Using siRNA's, we found that Akt1, but not Akt2, is responsible for phosphorylation of Skp2 at Ser72, and in turn, modulation of its protein stability. Furthermore, we demonstrated that when overexpressed in 293T cells, human Skp2 specifically interacts with endogenous Akt1, but not Akt2. Although the precise mechanism by which Akt1 can, whereas Akt2 cannot, signal to Skp2 has yet to be defined, and likely mechanisms include the localization of distinct Akt isoforms in cells and tissues. It is plausible that the nuclear localization of Akt1 which has been observed in some cell lines may allow it to interact with nuclear Skp2 and promote nuclear export, and that the more cytoplasmic localization of Akt2 may restrict its accessibility to Skp2. Although these and other possibilities have yet to be tested, our data is consistent with the recent finding that only Akt1 promotes G1 progression, DNA synthesis and proliferation of C2 myoblasts, whereas Akt2 is primarily required for exit from mitosis [38].

## Is Skp2B a better substrate for Akt?

**Figure 2:**
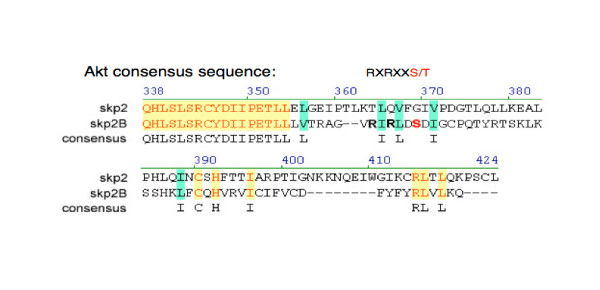
**Sequence alignment of the Skp2 and Skp2B proteins reveals that there is an additional potential Akt phosphorylation site at the C-terminus of the Skp2B protein**.

Skp2 cytoplasmic localization has been observed in many clinical tumor samples and is correlated with aggressive malignancy and poor diagnosis [15,57-60]. Our results offer a molecular mechanism for the cytoplasmic localization of Skp2. Furthermore, since elevated Akt also inactivates the Bad, Caspase-9 and FOXO proteins to allow tumor cells to evade the apoptosis pathway, cancer cells with cytoplasmic Skp2 localization tend to be more advanced. Recently, another novel Skp2 splicing isoform (Skp2B) was identified, which possesses many distinct molecular properties because it differs from the Skp2 protein at the carboxyl-terminus [61]. One major difference is that in contrast with the nuclear localization of Skp2, Skp2B localizes to the cytoplasm [58]. Comparing the Skp2 and Skp2B sequences showed that Skp2B contains the identified NLS, and does not have an obvious nucleus export signal (NES) sequence at its unique carboxyl-terminus. However, Skp2B contains an additional high probability Akt site in this region (Figure 2). This might make Skp2B an extremely effective strong substrate for Akt, which could result in cytoplasmic translocation by unknown mechanism.

## Concluding remarks

Collectively, we and others provide evidence that Akt directly controls Skp2 stability and oncogenic activity. We identified one major Akt phosphorylation site on Skp2 at Ser72 that is located within a putative Nuclear Localization Sequence (NLS). We demonstrated that phosphorylation of Ser72 by Akt1, but not Akt2, promotes Skp2 cytoplasmic translocation, likely due to a disruption of the NLS. This finding provides an explanation for previous observations whereby cytoplasmic Skp2 staining is detected in tissues from advanced breast and prostate cancer. Thus in cells released from serum-starvation, elevated Akt activity in the early G1 phase protects Skp2 from constitutive degradation by Cdh1. Hence, our finding expands our knowledge of how specific kinase cascades influence proteolysis governed by the APC/Cdh1 complex. In addition, these findings provide insight into how the activated PI3-K/Akt pathway leads to elevated Skp2 expression and subsequent enhanced p27 destruction in human cancers, providing further evidence that elevated Akt activity and cytoplasmic Skp2 expression may be causative for breast and prostate cancer progression. These results may provide a rationale to develop specific Akt1 inhibitors as efficient anti-cancer drugs.

## Competing interests

The authors declare that they have no competing interests.

## Authors' contributions

WW drafted the manuscript. DG designed the figures. All authors read and approved the final manuscript.
